# Neoatherosclerosis development following bioresorbable vascular scaffold implantation in diabetic and non-diabetic swine

**DOI:** 10.1371/journal.pone.0183419

**Published:** 2017-09-12

**Authors:** Nienke S. van Ditzhuijzen, Mie Kurata, Mieke van den Heuvel, Oana Sorop, Richard W. B. van Duin, Ilona Krabbendam-Peters, Jurgen Ligthart, Karen Witberg, Magdalena Murawska, Brett Bouma, Martin Villiger, Hector M. Garcia-Garcia, Patrick W. Serruys, Felix Zijlstra, Gijs van Soest, Dirk-Jan Duncker, Evelyn Regar, Heleen M. M. van Beusekom

**Affiliations:** 1 Department of Cardiology, Thoraxcenter, Cardiovascular Research school COEUR, Erasmus University Medical Center, Rotterdam, The Netherlands; 2 Department of Biostatistics, Erasmus University Medical Center, Rotterdam, The Netherlands; 3 Wellman Center for Photomedicine, Massachusetts General Hospital, Harvard Medical School, Boston, Massachusetts, United States of America; 4 Cardialysis B.V., Rotterdam, The Netherlands; 5 Dept. of Cardiovascular Surgery, University Hospital Zurich, Zurich, Switzerland; Queen Mary University of London Faculty of Medicine and Dentistry, UNITED KINGDOM

## Abstract

**Background:**

DM remains a risk factor for poor outcome after stent-implantation, but little is known if and how DM affects the vascular response to BVS.

**Aim:**

The aim of our study was to examine coronary responses to bioresorbable vascular scaffolds (BVS) in swine with and without diabetes mellitus fed a ‘fast-food’ diet (FF-DM and FF-NDM, respectively) by sequential optical coherence tomography (OCT)-imaging and histology.

**Methods:**

Fifteen male swine were evaluated. Eight received streptozotocin-injection to induce DM. After 9 months (M), 32 single BVS were implanted in epicardial arteries with a stent to artery (S/A)-ratio of 1.1:1 under quantitative coronary angiography (QCA) and OCT guidance. Lumen, scaffold, neointimal coverage and composition were assessed by QCA, OCT and near-infrared spectroscopy (NIRS) pre- and/or post-procedure, at 3M and 6M. Additionally, polarization-sensitive (PS)-OCT was performed in 7 swine at 6M. After sacrifice at 3M and 6M, histology and polymer degradation analysis were performed.

**Results:**

Late lumen loss was high (~60%) within the first 3M after BVS-implantation (P<0.01 FF-DM vs. FF-NDM) and stabilized between 3M and 6M (<5% change in FF-DM, ~10% in FF-NDM; P>0.20). Neointimal coverage was highly heterogeneous in all swine (DM vs. NDM P>0.05), with focal lipid accumulation, irregular collagen distribution and neointimal calcification. Likewise, polymer mass loss was low (~2% at 3M, ~5% at 6M;P>0.20) and not associated with DM or inflammation.

**Conclusion:**

Scaffold coverage showed signs of neo-atherosclerosis in all FF-DM and FF-NDM swine, scaffold polymer was preserved and the vascular response to BVS was not influenced by diabetes.

## Introduction

Patients with diabetes mellitus (DM) are generally at risk for worse outcome after stent-implantation than patients without DM.[1] Due to the complex and multifactorial nature of the disease process, including metabolic abnormalities and vascular dysfunction, the vascular response to stent-implantation is generally impaired, complicating current stenting strategies.[2–4]

The everolimus-eluting bioresorbable vascular scaffold (BVS) may offer advantages. It elutes everolimus in the first 3 to 6 months after implantation, inhibiting excessive neointimal growth [5] and starts losing structural integrity 3 months after implantation,[5] potentially enabling vascular function restoration.[6]

Histology in healthy swine demonstrated that struts are covered at 28 days and resorbed around 3 years with minimal calcification and inflammation.[7, 8] In selected patients from the ABSORB Cohort A (BVS 1.0) and B (BVS 1.1) trials, excellent results for treatment of coronary artery lesions were observed.[9, 10] However, only 3%-20% of the study population suffered DM and no studies were performed in diabetic animals. Thus, little is known about the effect of DM on the vascular response to BVS. DM may cause inflammation, which could influence scaffold degradation by disregulated acid-base balance or body-temperature.[11]

Animal models reflecting the impact of atherosclerosis and DM can be useful, as they allow us to study vascular responses and scaffold degradation in a more complex setting.[12] Moreover, swine can be rendered diabetic and in combination with an atherogenic diet they develop atherosclerosis comparable to humans.[13] Scaffolds can be placed in coronary arteries and in-vivo sequential intracoronary imaging can be performed by optical coherence tomography (OCT), polarization-sensitive (PS)-OCT and near-infrared spectroscopy (NIRS). After sacrifice, histology and gel permeation chromatography (GPC) can be performed to assess scaffold coverage and degradation of the polymer.

We examined the mechanistic and morphological aspects of the coronary response to BVS1.1 in DM and non-DM swine fed a fast-food diet (FF-DM, FF-NDM respectively) using longitudinal intracoronary imaging and histology.

## Materials and methods

### Experimental design

The Erasmus MC Animal Ethics committee approved the study, performed in accordance with the Guide for Care and Use of Laboratory Animals.[14] Fifteen male [Yorkshire x Landrace] swine with an age of ~11 weeks and a body weight of ~30kg were included (see S1 File for a detailed description). DM was induced by streptozotocin (140mg/kg iv, single dose) in 8 randomly selected male crossbred swine.[15] During streptozotocin injection, the swine were anesthetized with intramuscular azaperone (2 mg/kg, Stressnil, Janssen, Tilburg, The Netherlands), followed by intravenous thiopental (15 mg/kg, Nesdonal, Rhone Merieux, Lyon, France). The swine were housed in metabolic cages and were fed two fast-food (FF) meals a day during which they had access to food for one hour. The FF-diet is a diet containing 10% sucrose, 15% fructose, 25% (swine) lard, 1% cholesterol and 0.7% sodiumcholate (bile salts). The food intake was monitored for each animal separately and titrated to maintain growth at ~1.5 kg/week.

After 9 months, all 8 FF-DM and 5 FF-NDM received single 3.0x18.0mm Absorb BVS1.1 implants in 2, and 2 FF-NDM received single Absorb BVS1.1 implants in all 3 coronary arteries to ensure an even amount of scaffolds in FF-DM (N = 16) and FF-NDM (N = 16) (see S1 File for details about the BVS1.1). One day prior to BVS1.1-implantation the swine received 300 mg acetylsalicylic acid and a loading dose of 300 mg clopidogrel (Plavix, Sanofi). After an overnight fast, the swine were sedated using ketamine/ midazolam (20 mg/kg / 1 mg/kg i.m.) and atropine (1mg/30kg i.m.). After induction of anesthesia with thiopental (15 mg /kg i.v.; Nesdonal, Aventis), the swine were connected to a ventilator that administered a mixture of oxygen and nitrous oxide (1:2 [vol/vol]). Vascular access was obtained with an 8F vascular sheath in the carotid artery, 10.000 IU heparin was administered initially and thereafter 5000 IU of heparin was administered every hour. Anesthesia was maintained using 0.5–2.5 vol% isoflurane (Florence, Abbott Laboratories) as guided by hemodynamics and pain reflexes to ensure adequate analgesia and sedation. Antibiotic prophylaxis was administered by an intramuscular injection of 8 mL 200 mg/mL procaine-benzylpenicillin and 250 mg/mL streptomycin. After BVS-implantation, all swine were treated with clopidogrel (75mg) and acetylsalicylic acid (300mg) daily, until the end of the study. The latter also functions as analgesia during the post-operative recovery.

**Fig 1:**
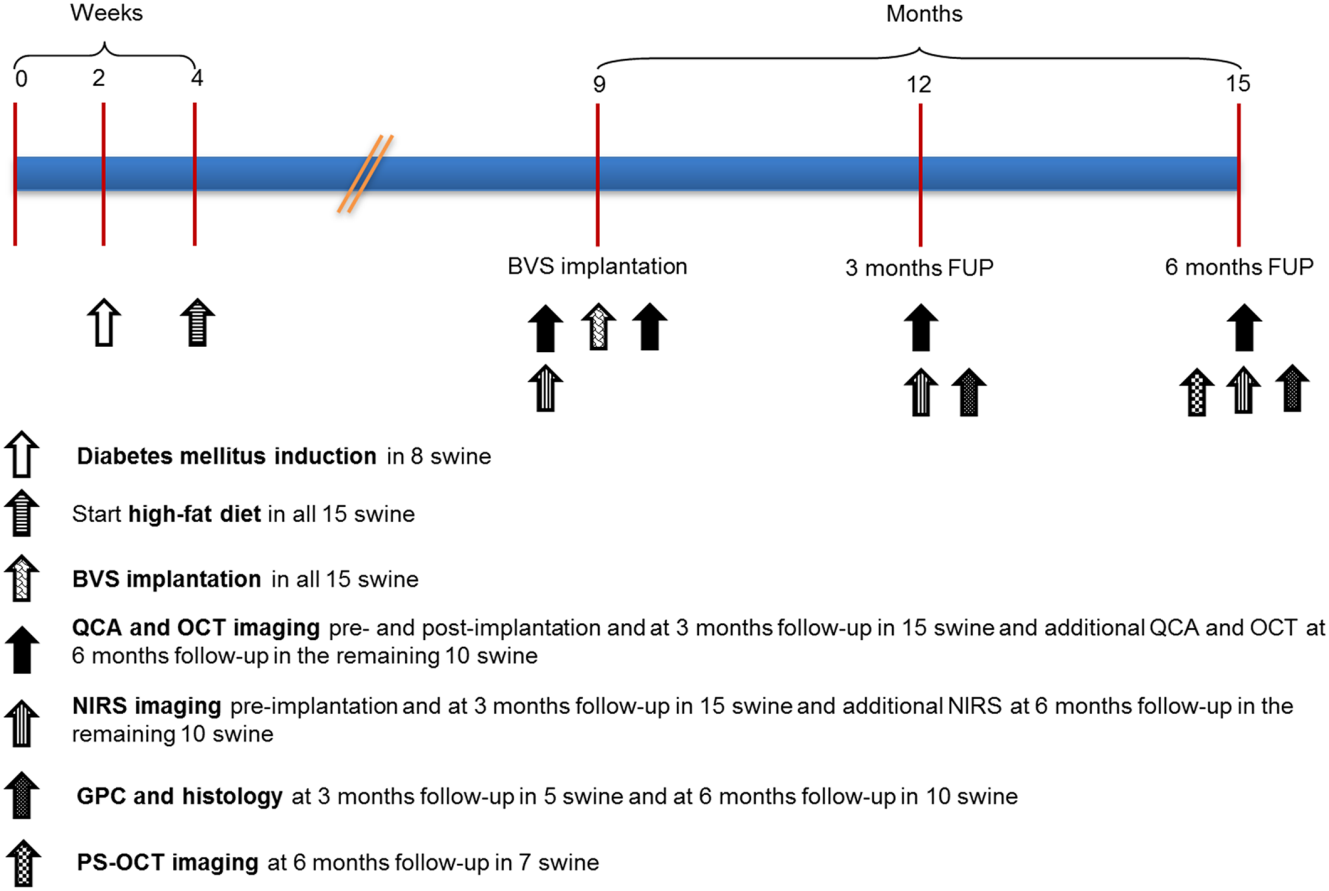
Study design. BVS = bioresorbable vascular scaffold, FUP = follow-up, QCA = quantitative coronary angiography, PS = Polarization Sensitive, OCT = optical coherence tomography, NIRS = near-infrared spectroscopy, GPC = gel permeation chromatography.

Sequential coronary imaging included QCA and OCT pre-, immediately, 3 and 6 months (M) post-implantation and NIRS pre-, 3M and 6M post-implantation. PS-OCT was performed in N = 3 FF-DM and N = 4 FF-NDM BVS at 6M. 3M imaging was included when pre and/or post-implantation imaging were available and 6M imaging was included when pre- and/or post—and 3M imaging were available. After the 3M imaging assessment, 3 FF-DM and 2 FF-NDM were sacrificed and after the 6M imaging assessment the remaining swine were sacrificed. After sacrifice, hearts were removed, the coronary tree dissected free and coronary arteries containing BVS randomized to histological (3M N = 5/10, 6M N = 12/22) or GPC analysis (3M N = 5/10, 6M N = 10/22) (Fig 1).

Fasting blood samples were obtained at baseline, 3M and 6M to measure glucose, total, low- and high-density lipoprotein cholesterol (TC, LDL, HDL) and triglyceride levels. Furthermore, in the FF-DM swine, glucose levels were assessed weekly by 24-hour urine samples. When glucose appeared in undiluted urine samples, venous glucose and ketone levels were checked via ear vein puncture and a handheld reader. When glucose levels were high (>20 mmol/L), in combination with ketone production, subcutaneous insulin (approx. 5–15 units once daily) was given to eliminate detectable ketone production while maintaining hyperglycemic state.

### In-vivo QCA, (PS)-OCT and NIRS analysis

See S1 File for a detailed description of the imaging analyses. Coronary angiograms were obtained in two orthogonal views and QCA-analysis was performed (CAAS, version 5.9.2 Pie Medical Imaging BV). Mean (LD) and minimal lumen diameter (MLD), scaffold to artery (S/A) ratio, acute gain and late lumen loss (LL) were documented. Longitudinal matching of OCT pullbacks (C7XR Fourier-Domain, St. Jude Medical) was performed as described previously using dedicated CURAD analysis software (CURAD BV).[16]

OCT parameters for vascular reaction, including lumen and scaffold dimensions and scaffold strut appearance, apposition, neointimal coverage and coverage morphology were assessed in 1-mm intervals using off-line OCT analysis software according to previously published methodology.[17] In pre-implantation lesions not exceeding the penetration depth of OCT, plaque burden (PB) was determined. Mean number of discernible struts were documented immediately post-implantation and at follow-up. Changes in strut appearances were categorised as preserved, open, dissolved bright, and dissolved black box.[18] Struts were scored as covered or uncovered and the morphology of the coverage—defined as [SA—LA][16]–was described as homogeneous or heterogeneous. Heterogeneous coverage was furthermore described as lipid-laden, calcified, surrounding the struts or subluminal, or mixed (S1 Fig).[19]

PS-OCT was performed using a prototype imaging system. PS-OCT provides a measure of tissue birefringence, an optical tissue property that describes the interaction with polarized light. It grossly relates to microscopic tissue organization, and enables characterization of collagen content and smooth muscle cell (SMC) density in atherosclerotic plaques.[20]

NIRS analysis (LipiScan, InfraReDx) was used for lipid core plaque (LCP) characterization.[21] Lipid-core burden index (LCBI) was documented, indicating high probability that LCP is present. To evaluate the agreement between OCT and NIRS for detection of lipid, we compared—per scaffold—the LCBI score to the percentage of OCT cross-sections with lipid-containing morphology.

### Ex-vivo degradation analysis

GPC was performed as described previously (see S1 File).[7] Degradation in our model was studied in relation to DM, time, inflammation, scaffold recoil, OCT-derived strut appearance and pre-implantation plaque burden.

### Ex-vivo histological analysis

See S1 File for a detailed analysis. Proximal, middle and distal sections within each BVS were obtained. Tissue sections were stained by Hematoxylin-Eosin (HE) as an overview stain, Resorcin-Fuchsin for elastin, Alcian-Blue for proteoglycans, Oil-red-O (ORO) for lipids, Picrosirius Red (PSR) for collagen, von Kossa for calcium, and immunohistochemistry for smooth muscle cells (aSMA, clone 1A4, Dako, the Netherlands) and leukocytes (CD45, clone MCA 1447, AbD Serotec, UK). Polarization microscopy was performed to assess scaffold struts.

Histological analysis included neointimal healing and organization, collagen distribution, injury and inflammation score, lipid accumulation and presence of calcium classified as subluminal or surrounding struts (S2 Fig).

### Statistical analysis

Statistical analysis (SPSS 20.0) entailed the Kolmogorov-Smirnov test for normality of the data. Normal distribution was expressed as mean ± standard deviation. Non-normally distributed data were presented as median with interquartile range. Comparison of in-vivo imaging between FF-DM and FF-NDM was performed by generalized estimating equations (GEE) modeling. GEE is a statistical method that accounts for the clustered nature of >1 scaffold analyzed from one swine, which might result in unknown correlations among measurements within these scaffold clusters. A linear response model was applied with an exchangeable structure for the within-cluster correlation matrix. For repeated measures, GEE modeling was performed using a linear response model with an autoregressive (AR(1)) structure for the within-cluster correlation matrix. Comparison of ex-vivo GPC between FF-DM and FF-NDM swine was performed by independent samples t-test. To assess variable relations, the Spearman correlation coefficient was computed. All statistical tests were 2 tailed, and P<0.05 was considered statistically significant.

## Results

### Plasma measurements

Average TC, LDL and HDL were similar between FF-DM and FF-NDM (TC 18.7±5.0mmol/l and 19.0±5.8mmol/l (P = 0.86); LDL 15.1±5.1mmol/l and 15.6±5.1mmol/l (P = 0.75); HDL 5.4±0.8mmol/l and 5.7±0.7mmol/l (P = 0.13) in FF-DM and FF-NDM respectively). In the 8 swine that received a steptozotocin-injection, DM was successfully induced. Average plasma glucose and triglyceride levels were elevated in FF-DM compared to FF-NDM (15.0±7.8mmol/l vs. 4.5±1.0 mmol/l (P<0.01), 1.1±0.8mmol/l vs. 0.6±0.5mmol/l (P = 0.02), respectively). All FF-DM swine received insulin in the first 10 weeks after streptozotocin injection and 2 FF-DM swine received insulin throughout the entire study based on detectable venous ketone body production.”

### In-vivo QCA, (PS)-OCT and NIRS

All 32 BVS were successfully implanted with a mean S/A-ratio of 1.1±0.1 in FF-DM and FF-NDM (P = 0.20).

**Fig 2:**
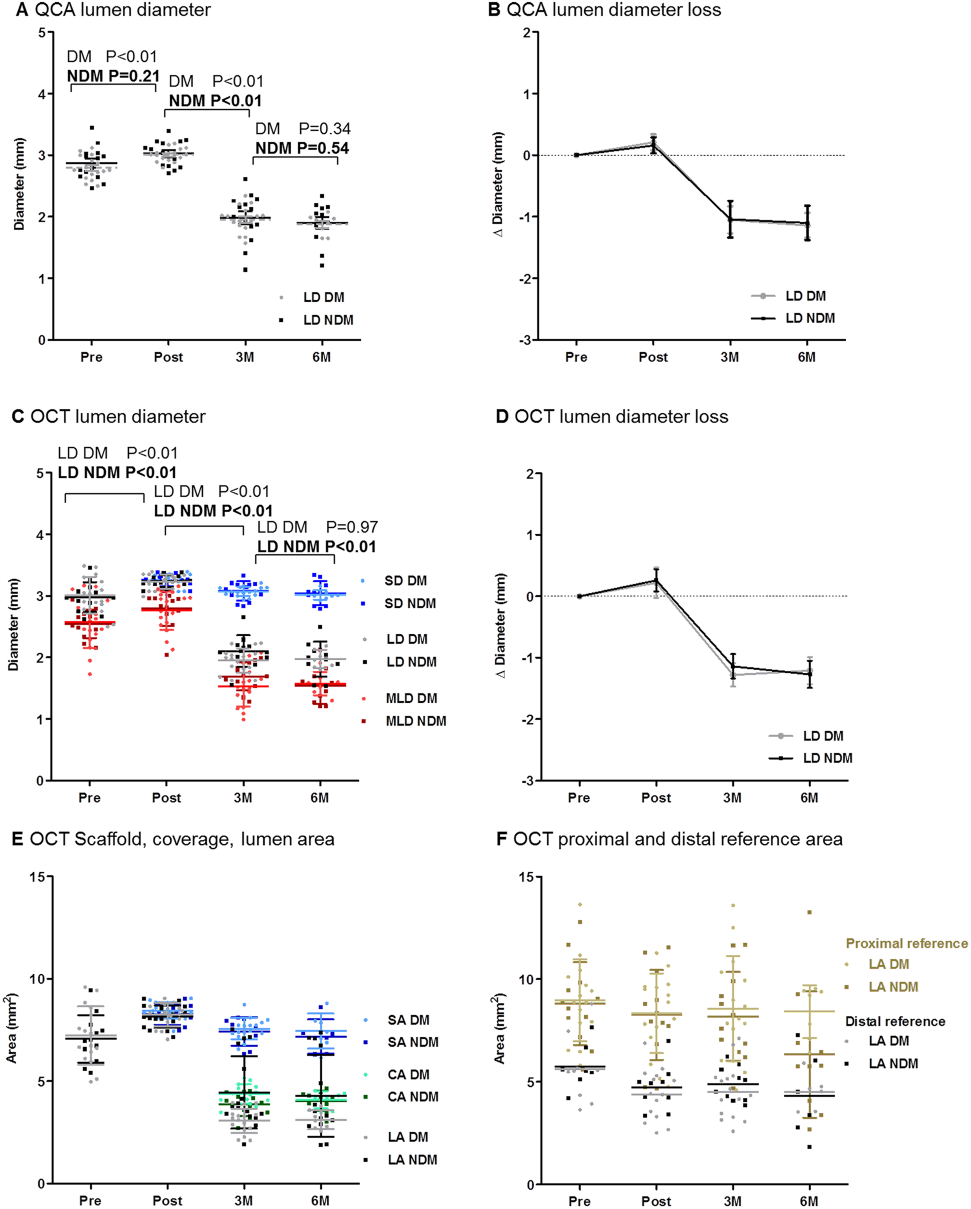
QCA and OCT analysis results. **A+B**) Mean lumen diameter (LD) slightly increased from pre-to post-implantation, decreased from post-implantation to 3M and remained stable from 3M to 6M. Grey: FF-DM, black: FF-NDM. **C-E**) Mean lumen area (LA), lumen diameter (LD) and minimal lumen diameter (MLD) increased slightly from pre- to post-implantation. Mean LA, LD and MLD and mean scaffold area (SA) and scaffold diameter (SD) decreased from post-implantation to 3M and mean LA, LD, MLD, SA, SD and coverage area (CA) remained stable from 3M to 6M. **F**) Proximal and distal reference LA slightly decrease from pre- to post-implantation and stabilized from post-implantation to 3M and 6M.

QCA findings are presented in Fig 2A + 2B and S1 Table. Pre-implantation lesions were mild and similar between FF-DM and FF-NDM (P = 0.33). In all swine, mean LD decreased from post-implantation to 3M (P<0.01) and remained fairly stable from 3M to 6M (P = 0.34 and P = 0.54, respectively).

Quantitative OCT findings are presented in Fig 2C–2F and S1 Table. Pre-implantation lumen dimensions (LD, LA and MLA) were similar between FF-DM and FF-NDM (P = 0.46, P = 0.46 and P = 0.74) and pre-implantation %PB was mild (9±2% FF-DM, 10±1% FF-NDM; P = 0.58). Scaffolds were implanted according to protocol with an S/A ratio ≥1.1. Thus, LD increased in all swine from pre- to post-implantation (P>0.10). No signs of scaffold damage nor procedure-related injury were documented. No edge dissection or thrombus was observed and minor tissue prolapse was documented. Mild acute ISA was observed in 2 FF-DM BVS (mean ISA area 0.26±0.11mm^2^) and 1 FF-NDM BVS (mean ISA area 0.03mm^2^; P<0.01). From post-implantation to 3M, mean LD, LA and MLA decreased (~60%). At 3M, all ISA resolved, no late acquired ISA developed and all struts were covered. Restenosis, however, hampered OCT imaging at 3M in one FF-NDM. At 6M, OCT demonstrated a substantial neointima with a highly heterogeneous morphology, which was confirmed by histology (S3 Fig). From 3M to 6M, mean LD, LA, MLA, SD and SA remained fairly stable in all swine (Fig 2C–2E; S1 Table)

#### Changes in coverage morphology

See Table 1 for OCT findings. A heterogeneous morphology predominated in all swine at 3M, with a relatively high prevalence of calcium (25% (16%; 43%) in FF-DM and 16% (5%; 36%) in FF-NDM; P = 0.49). At 6M, the heterogeneous pattern predominated and was mainly characterized by calcium (41% (33%; 66%) in FF-DM and 59% (37%; 85%) in FF-NDM) (P = 0.82) (S1 and S2 Videos). Moreover, the accumulation of calcium increased (P<0.05), whereas the accumulation of lipid remained moderate (FF-DM P = 0.67, FF-NDM P = 0.97).

**Table 1:** OCT coverage analysis.

	3M			6M			P‡	
	FF-DM	FF-NDM	P*	FF-DM	FF-NDM	P*	FF-DM	FF-NDM
Coverage thickness, mm	0.42±0.08	0.39±0.11	0.50	0.39±0.06	0.41±0.11	0.59	0.05	0.38
Coverage area, mm^2^	4.38±0.48	4.03±0.67	0.19	4.09±0.42	4.20±0.59	0.65	0.03	0.10
**Coverage appearance**								
Homogeneous, %	0 (0;6)	0 (0;4)	0.17	0 (0;0)	0 (0;4)	0.51	0.05	0.09
Heterogeneous, %	100 (94;100)	100 (96;100)	0.17	100 (100;100)	100 (96;100)	0.51	0.05	0.09
Lipid-laden, %	0 (0;12)	0 (0;14)	0.57	6 (6;11)	6 (0;15)	0.97	0.54	0.79
Calcified, %	25 (16;43)	16 (5;40)	0.49	41 (33; 66)	72 (37;87)	0.64	0.01	<0.01
*Strut area*, *%*	19 (10;31)	13 (0;34)	0.70	29 (13;63)	15 (5;47)	0.49	0.14	0.05
*Subluminal*, *%*	3 (0;7)	0 (0;4)	0.51	5 (0;6)	3 (0;29)	0.29	0.49	0.07
*Strut area + subluminal*, *%*	0 (0;0)	0 (0;0)	0.55	5 (0;8)	19 (3;29)	0.11	0.09	<0.01
Mixed, %	0 (0;7)	0 (0;9)	0.99	22 (12;48)	13 (1;40)	0.71	<0.01	0.04

Normally distributed data are presented as mean±SD, non-normally distributed data as median (interquartile range). FF-DM = fast-food fed diabetic swine, FF-NDM = fast-food fed non-diabetic swine, OCT = optical coherence tomography, 3M = 3 months follow-up, 6M = 6 months follow-up.

*P-value for the comparison between FF-DM and FF-NDM swine,

^‡^P-value for the difference between 3M and 6M.

**Fig 3:**
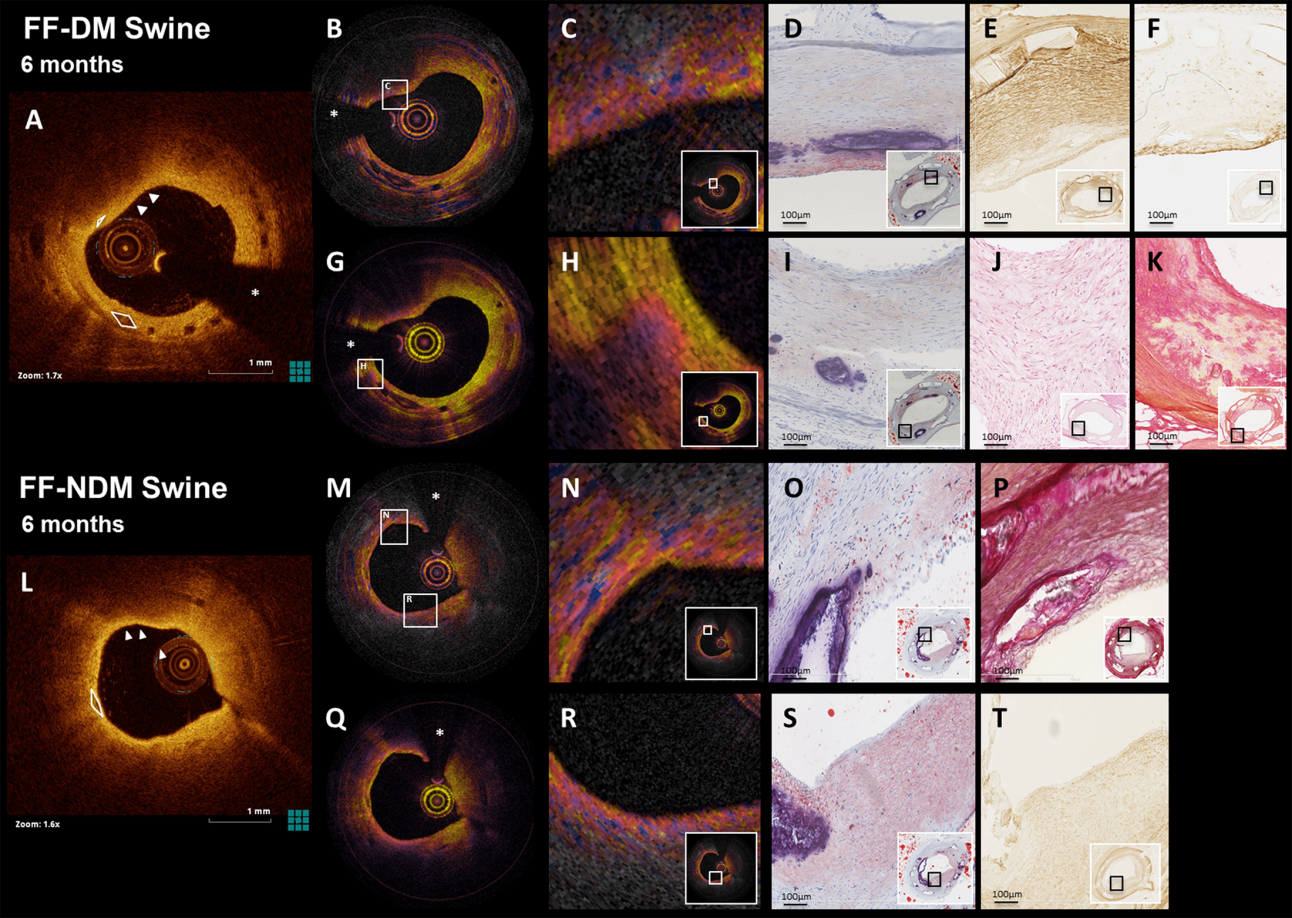
(PS)-OCT and corresponding histology at 6M. OCT demonstrated the development of a highly heterogeneous neointima with lipid and calcium accumulation in FF-DM and FF-NDM swine at 6 months (**A, L**), which was confirmed by histology (**D, I, O, S**; Oil-red-O). Phase retardation corresponding to tissue birefringence (**B, M**) and depolarization (**G, Q**) imaged by PS-OCT, demonstrated enhanced birefringence and depolarization (**C**) in an SMC-poor area (**E**; aSMA) with inflammation (**F**; CD45). Furthermore, PS-OCT demonstrated focal depolarization (**H**) in a collagen-poor area with loss of structure and evidence of early necrosis (**J, K**; HE, PSR). **N** shows coarse-grained high birefringence in an area with strongly circumferentially organized intimal SMCs (**P**; RF); lipid-rich, SMC-poor tissue (**T**; SMA) exhibits a more finely speckled heterogeneity, associated with a rapid loss of polarization degree (**R**). Asterisk (*) indicates guidewire artefact, arrowheads lipid, white lines calcium.

PS-OCT was only qualitatively analyzed and showed a heterogeneous neointima with spots of elevated birefringence (Fig 3E and 3K). Rapid depolarization of the signal was often observed, focally (Fig 3F2) and in areas with the appearance of lipid-rich plaque and inflammation (Fig 3F and 3L).

See S2 Table for NIRS findings. Similar to OCT, the prevalence of lipid was relatively low in FF-DM (9/15 BVS) and FF-NDM (3/11 BVS) at 3M with relatively low LCBI scores (3.00 (0.00; 22.50) in FF-DM, 0.00 (0.00; 3.00) in FF-NDM; P = 0.69). From 3M to 6M, the prevalence of lipid increased numerically, in FF-DM (6/6 BVS) and FF-NDM (5/8 BVS). Subsequently, LCBI scores slightly increased (17.50 (9.75; 26.00) in FF-DM, 6.50 (0.00; 47.25) in FF-NDM; P = 0.49).

The association of LCBI-score and percentage of OCT cross-sections with a lipid-laden or mixed appearance was modest at 3M (Spearman’s rho 0.397; P = 0.05) but became stronger at 6M (Spearman’s rho 0.666; P<0.01).

#### Changes in strut appearance

See S3 Table. The number of discernible struts per OCT cross-section was similar post-implantation (8±2 in FF-DM, 8±1 in FF-NDM) and at 3M (8±1 in FF-DM, FF-NDM) and decreased to 6M (7±1 in FF-DM, 6±2 in FF-NDM). The majority kept a preserved box appearance from 3M (79% in FF-DM, 81% in FF-NDM) to 6M (77% in FF-DM, 68% in FF-NDM). Interestingly, a substantial amount of struts appeared as dissolved black box at 3M (20% in FF-DM, 18% in FF-NDM) and 6M (22% in FF-DM, 31% in FF-NDM).[22]

### Ex-vivo GPC

See Table 2. The initial Mn, Mw and PDI were 109.55 KDa, 229.68 KDa and 2.10 respectively. The initial mass was 8.53±0.08mg in FF-DM and 8.50±0.07mg in FF-NDM. Up to 6M, Mn, Mw and PDI decreased and mass loss was low: 5.5±1.9% in FF-DM and 4.3±1.4% in FF-NDM (P = 0.28).

**Table 2:** GPC and histology results.

	3M			6M		
	FF-DM	FF-NDM	P*	FF-DM	FF-NDM	P*
**GPC results**						
BVS evaluated, n	3	2		5	5	
Mn, KDa	82.76±4.36	80.13±0.85	0.41	67.17±3.11	65.55±3.40	0.46
Mw, KDa	159.31±7.07	160.39±4.03	0.84	134.55±2.47	132.23±8.03	0.56
PDI	1.93±0.02	2.00±0.03	0.10	2.01±0.09	2.02±0.04	0.84
%Mass loss	2.6±0.6	2.0±6.6	0.86	5.5±1.9	4.3±1.4	0.28
**Histological results**						
BVS evaluated, n	3	2		5	7	
Neointimal thickness, mm	0.76±0.08	0.62±0.07	0.13	0.60±0.06	0.65±0.11	0.38
Medial thickness, mm	0.06±0.01	0.06±0.03	1.00	0.07±0.02	0.09±0.03	0.17
Adventitial thickness, mm	0.13±0.02	0.14±0.09	0.95	0.10±0.02	0.11±0.03	0.32
Scaffold area, mm^2^	7.20±0.36	6.53±0.97	0.50	5.61±0.73	5.65±0.63	0.92
Injury sore	1.30±0.42	1.10±0.14	0.58	1.10±0.13	1.04±0.33	0.74
Inflammation score	0.95±0.32	0.32±0.08	0.08	0.22±0.18	0.30±0.47	0.71
Lipid accumulation, %	7.2 (5.4;17.3) 3.72.8;5.3)	15.8 (9.5;20.0) (9.0;(0.9;1.4)	0.83	19.5 (15.8;19.8)	9.4 (6.1;24.6)	0.76
Calcium						
*Surrounding strut*, *%*	88.03±8.21	94.05±8.41	0.48	72.54±11.63	74.21±19.63	0.87
*Score per-strut*	1.23±0.24	1.20±0.14	0.87	0.86±0.38	0.94±0.26	0.67
*Subluminal*, *n*	2	1	0.79	3	6	0.36

GPC = gel permeation chromatography, Mn = number average molecular weight, Mw = weight average molecular weight, PDI = polydispersity index [Mn/Mw]. Percentage (%) mass loss = [(Initial mass prior to scaffold implantation [T = 0] (mg)–Found mass (mg)) / Initial mass [T = 0] (mg) x 100].

*P-value for the comparison between FF-DM and FF-NDM swine. Footnotes and the remaining abbreviations are as listed in Table 1.

There was no relationship between scaffold degradation and DM (P>0.10), or with OCT-derived pre-implantation %PB (P = 0.22), scaffold recoil (P = 0.59), preserved (P = 0.92), open (P = 0.45) or dissolved black box appearance (P = 0.99) at 3M, or at 6M (P = 0.29, P = 0.73 P = 0.27, P = 0.36 and P = 0.64 respectively).

### Ex-vivo histology

See Table 2. All struts were covered by neointima 3M post-implantation (FF-DM 0.76±0.1mm, FF-NDM 0.62±0.1mm; P>0.10). Neointimal organization score was higher at 3M than 6M (P<0.01) in FF-DM and FF-NDM (P = 0.14). Injury was moderate. Injury scores were 1.30±0.42 and 1.10±0.14 (P = 0.58) at 3M and 1.10±0.13 and 1.04±0.33 (P = 0.74) at 6M in FF-DM and FF-NDM, respectively.

**Fig 4:**
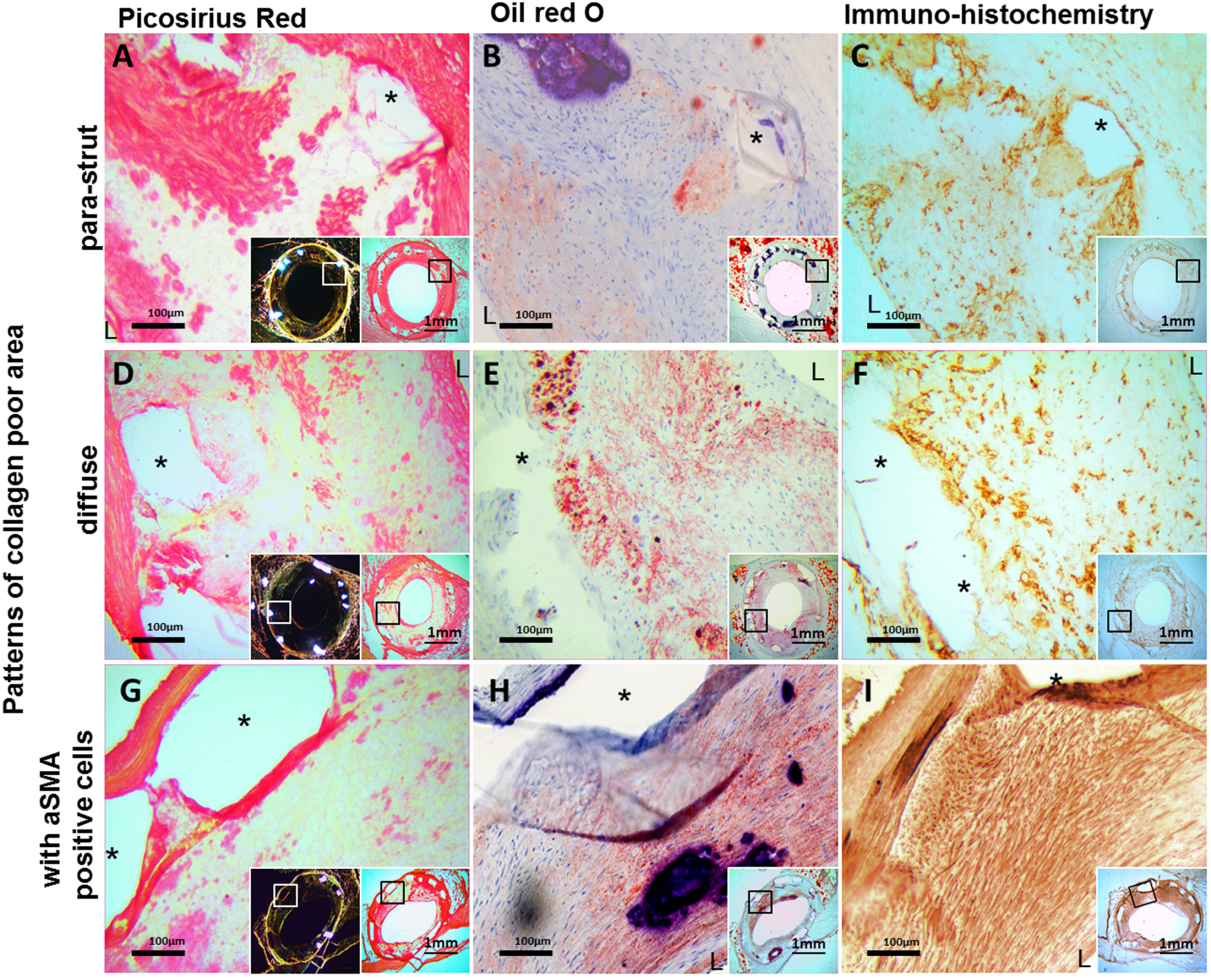
Irregular collagen distribution in the neointima. Collagen poor areas in peri-strut regions and neointima (**A, D**; Picrosirius Red) often demonstrated lipid accumulation (**B, E**; Oil-red-O), and leucocytes (**C, F**; CD45). Additionally, **G** (Picrosirius Red) demonstrates a patchy collagen poor lesion with lipid accumulation (**H**; Oil-red-O) and smooth muscle cells (**I**; aSMA). *: strut void, **L**: lumen.

Collagen poor regions were observed within the neointima. They contained leukocytes, were evident at sites with extracellular lipids and often coincided with calcifications (Fig 4). Collagen poor but SMC positive tissue generally contained lipid accumulation (P>0.10 for FF-DM vs. FF-NDM) (Fig 4G–4I). Mainly intra- and extracellular lipid deposits with few cholesterol crystals were observed and advanced necrotic cores were absent.

**Fig 5:**
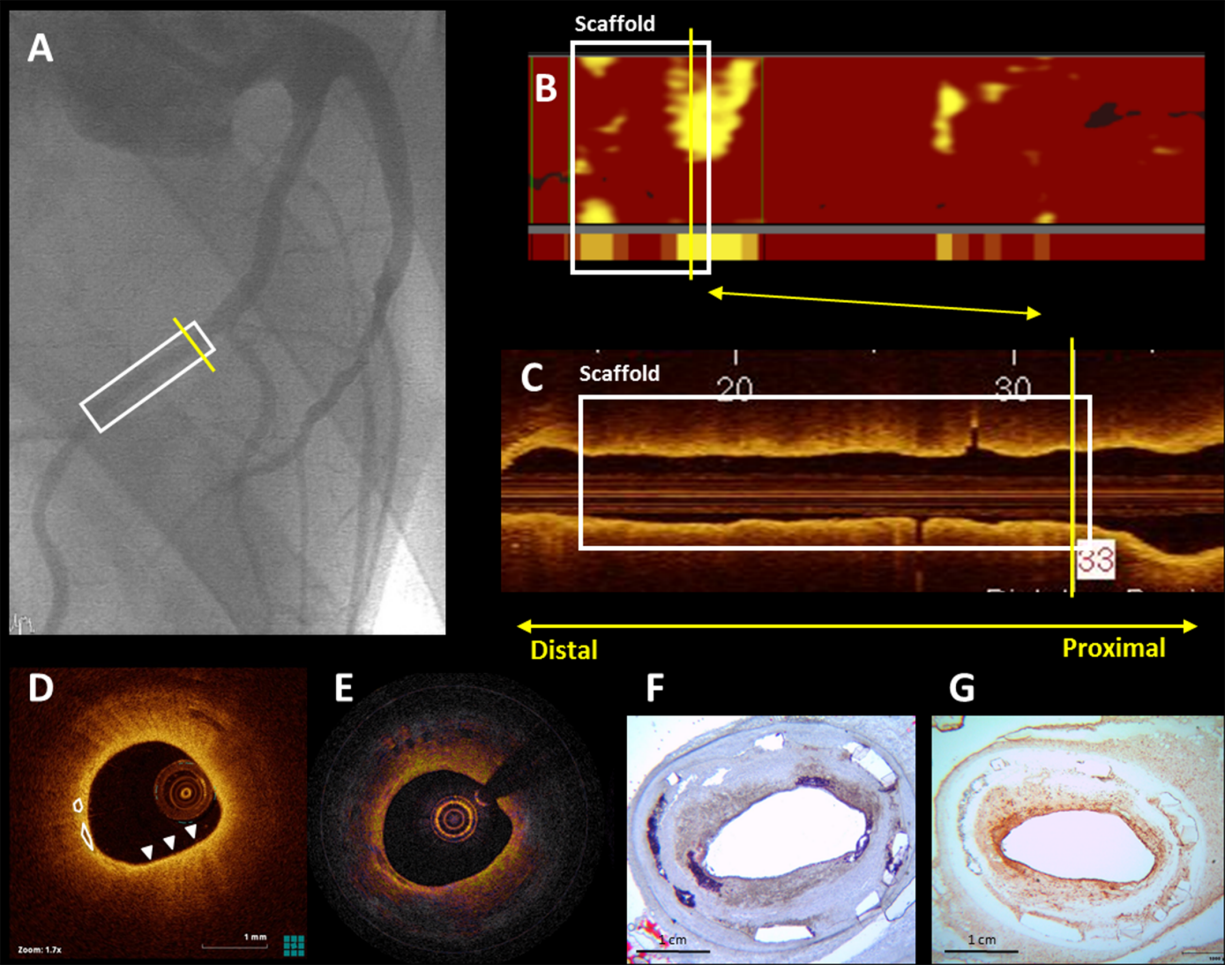
Corresponding QCA, OCT, NIRS and histology at 6M. QCA (**A**) demonstrates the scaffolded region (white block), with the yellow line indicating the region corresponding to the NIRS, (PS)-OCT and histology images (**B-G**). The NIRS chemogram demonstrates presence of lipid (**B**), also observed by OCT (**D**; arrowheads) that additionally demonstrates the presence of calcium (white circles). The PS-OCT phase retardation image demonstrates a finely grained pattern (**E**) consistent with lipid-rich neointima (**F**; Oil-Red-O) and active inflammation (**G**; CD45).

Neointimal and peristrut calcifications were observed in FF-DM and FF-NDM **(P = 0.04)** at 3M and 6M (Fig 3, Table 2), with varying size, shape and location between animals, suggesting an inter-animal difference. From 3M to 6M, lipid-accumulation remained (Fig 5) and calcifications were observed more frequently subluminally (in 3/5 FF-DM BVS, 6/7 FF-NDM BVS).

At 6M, signs of myxoid degeneration with lipid accumulation were present (S4 Fig). In 3 BVS (N = 1 FF-DM, N = 2 FF-NDM) thrombus remnants were observed at 6M but not at 3M.

Furthermore, the polymeric scaffold struts demonstrated birefringence with polarized light, confirming preservation of scaffold struts in all swine.

## Discussion

The present study describes the coronary artery response to BVS1.1 in FF-DM and FF-NDM swine. A remarkable neotintima burden with a highly heterogeneous appearance was observed in all swine, with lipid accumulation and calcification, indicative for the formation of neoatherosclerosis. The scaffold polymer was preserved up to 6M, independent of inflammation or the presence of DM in FF-swine.

### Neoatherosclerosis development following BVS-implantation

Interestingly, considerable neointima formation with complete strut coverage was observed in all swine, independent of the presence of DM. This is relevant, as uncovered struts have been associated with adverse events like stent thrombosis.[23] However, the neointima was highly heterogeneous in all swine, with substantial lipid- and calcium accumulation and lack of intimal organization at 6M, consistent with neoatherosclerosis formation. This is of note, as previously published experimental studies in healthy swine and clinical studies in selected patients demonstrated a favorable vascular response with rather homogeneous coverage following BVS-implantation.[7, 10, 24] There are three main differences between our study and the previously published experimental study by Onuma et al., namely 1: the version of the BVS; 2: the species in which the majority of the study was performed (miniswine) and 3: the presence of diabetes and hypercholesterolemia; In addition, we expect that, given the size of the animals in our study, our stents were implanted more distally.

First, in the histological evaluation of the first-generation BVS, revision 1.0, implanted in the coronary arteries of healthy Yorkshire x Landrace or Yucatan miniswine of unspecified age, minor calcifications were observed around the scaffold struts in the majority of BVS as early as 28 days post-implantation.[7] After an initial increase, the presence of calcifications decreased to 17.2% between two and four years post-implantation. In our study, implantation of BVS 1.1 demonstrated calcifications around the majority of scaffold struts at 3M, which included calcifications of the luminal border at 6M in both FF-DM and FF-NDM swine. Second, the majority of swine coronary arteries studied by Onuma et al. were from Yucatan miniswine, whereas we studied the coronary arteries of Yorkshire x Landrace swine with a similar race as the 28-day Onuma data, showing calcifications as early as 28 days. Although strain differences have not been described before as cause of a different vascular response to the polymer, this cannot be excluded as a cause for the different vascular response observed between the study by Onuma et al. and our study. The final main difference between the study by Onuma et al. and our study is the presence of hypercholesterolemia in all animals, and of DM in a subgroup, where especially the former seems to drive the response for the vascular response. Hypercholesterolemia is indeed detrimental to the endothelium, diminishing the vascular wall barrier-function against excessive uptake of circulating lipids, resulting in neoatherosclerosis formation.[25, 26] In humans, calcification is accelerated by young age and mechanical stress and this might explain why we found such a high incidence in our swine model.[27] While it is not completely clear to what extent our observations can be extrapolated to the clinical setting, our observation of considerable neoatherosclerosis formation under diet induced dyslipidemia might point at neoatherosclerosis as an important contributor to BVS failure at long term, similar to that described for DES and BMS.[28, 29, 30] It might also be in line with individual observations of asyptomatic neoplaque rupture after BVS-implantation and recently reported cases of BVS thrombosis.[10, 17, 24, 31]

Furthermore interesting is the fact that neoatherosclerosis development was observed in all swine, independent of the presence of DM. Kereiakes et al. demonstrated that diabetic patients who were receiving insulin treatment had a worse outcome after stent-implantation compared to those who were not receiving insulin.[32] In our study we did not observe a correlation between the amount of insulin given and the development of neoatherosclerosis. Of note, the swine receiving the most insulin, were not the swine that developed the worst neoatherosclerosis. Even in the two swine that received insulin throughout the entire study, who also received the greatest amount of food to ensure a similar growth pattern in all swine, neoatherosclerosis development was similar compared to all other FF-DM and FF-NDM swine. Factors such as duration of DM and hypertension may attribute to the severity of atherosclerotic disease and may therefore attribute to a more human-like evaluation of the coronary vascular healing response after BVS-implantation. Moreover, hypertension, not present in the current study, has been associated with adverse atherosclerosis-related events in DM patients.[33] Future studies assessing the coronary healing response after stent or scaffold-implantation in swine should consider using mature swine, and include risk factors such as hypertension to accurately evaluate the coronary vascular healing response to stent-implantation in a model that mimics human coronary atherosclerotic disease.

This study presents the first data of neoatheroasclerotic tissue organization characterized with PS-OCT. We observed enhanced tissue birefringence in areas with SMC alignment in the neointima, as well as in areas with inflammation. Macrophage recruitment in atherosclerosis has been associated with formation of cholesterol crystals [34], which are highly birefringent [35]. The contrast provided by PS-OCT, consisting of birefringence and depolarization, reflects tissue organization, which has an impact on structural plaque stability. A fuller understanding of the features highlighted by PS-OCT may complete our comprehension of neoatherogenesis and its impact on clinical sequelae.

### Preserved scaffold integrity

GPC and histology demonstrated preserved scaffold integrity up to 6M after BVS1.1-implantation in all swine, which was not affected by DM or inflammation. This is expected as the scaffold starts losing structural integrity at 3–6 months, and scaffold resorption is driven by hydration, rather than inflammation. Although, theoretically, other factors associated with inflammation such as deregulated acid-base balance or body-temperature could influence scaffold degradation, this was not seen in the present study.[11]

Interestingly, OCT did demonstrate morphological changes at individual strut levels despite preserved scaffold integrity. The OCT classification of strut appearances was developed in the ABSORB Cohort A trial to characterize the optical changes of the struts during the process of bioresorption.[9] However, preclinical evaluation of BVS1.0 demonstrated full degradation of the scaffold struts by GPC, while OCT demonstrated the presence of so-called ‘preserved black boxes’ within the vascular wall.[7] As the OCT signal is arising from the interface of structures with different optical indices, OCT reflects changes of tissue surrounding the struts, rather than changes in strut morphology. This should be kept in mind when interpreting in-vivo clinical and preclinical OCT observations in BVS.

### Methodological considerations

Sacrifice was planned for 1/3 of the swine at 3M, and thus the serial BL, 3M and 6M sample size was relatively small. The aim of our study, however, was to longitudinally examine mechanistic and morphological aspects of the coronary response to BVS1.1 in FF-DM and FF-NDM swine. To accurately assess the mechanistic aspects—e.g. scaffold resorption—at various time points, additional planned sacrifice at 3M was beneficial. Furthermore, atherosclerotic lesions that developed in FF-DM and FF-NDM swine before scaffold-implantation were relatively small. However, distribution and size of the lesions were similar in both groups, allowing for adequate comparison of vascular responses following BVS-implantation between FF-DM and FF-NDM swine.

## Conclusions

Scaffold coverage showed signs of neo-atherosclerosis in all FF-DM and FF-NDM swine, scaffold polymer was preserved and the vascular response to BVS was not influenced by diabetes.

## Supporting information

S1 Fig Qualitative OCT analysis of the scaffold coverage. In the top OCT cross-sections of a homogeneous, heterogeneous, lipid-laden, calcified and mixed appearance of the coverage are depicted and on the bottom the magnifications. The ‘open’ stars indicate the black boxes of the scaffold struts at follow-up. The asterix (*) indicates the guide wire artifact. The arrowheads indicate the region containing lipid and the drawn white lines indicate calcified regions.
(TIF)

S2 Fig Schematic representation of histological regions of interest. Within the neointima, 2 specific regions were discerned: para-strut neointima, defined as in contact with the struts, and subluminal: located near the lumen.
(TIF)

S3 Fig Restenosis at 3M. Restenosis of a BVS implanted in an FF-NDM swine at 3M. Coronary angiography post-implantation (A) and at 3M (B) demonstrates a significant lumen loss, with a percentage diameter stenosis (%DS) of 70% which persisted at 6M, (C). At 3M OCT was not performed as the lesion was considered too tight to allow passage of an OCT catheter without risk of causing ischemia and all the potential sequelae thereof. Therefore OCT was restricted to 6M follow-up, the scheduled sacrifice time point. OCT demonstrated a highly heterogeneous neointima (D), which is confirmed by histology demonstrating a large neointimal burden with calcification subluminal and surrounding the struts (E H&E + F, ORO). D Lesion = Diameter of the lesion, Ref D Lesion = Reference diameter.
(TIF)

S4 Fig Organized and non-organized luminal layers in the neointima. The vessels with well-organized neointimal layers (two arrows in **A-D**) showed dense elastic fibers (**B**) with 3 or more layers of αSMA positive cells (**C**) without lipid accumulation (**D**). The unorganized neointima showed myxoid degeneration (double arrow in **E-H**) with disarray and low density of αSMA positive cells (**G**). In the same area, lipid accumulation was clearly seen (**H**). αSMA: alpha smooth muscle cell actin, L: lumen, **A-D**: 3 months DM, **E-H**: 6 months non-DM, **A** and **E**: H&E, **B** and **F**: Resorcin-Fuchsin, **C** and **G**: αSMA, **E** and **H**: Oil red O, Scale bar in **A -H**: 100 μm, insert bar of **A** and **E**: 1000 μm.
(TIF)

S1 Table Quantitative QCA and OCT analysis results. Normally distributed data are presented as mean ± SD, non-normally distributed data as median (interquartile range). FF-DM = fast-food fed diabetic swine, FF-NDM = fast-food fed non-diabetic swine, QCA = Quantitative coronary angiography, OCT = optical coherence tomography, BVS = bioresorbable vascular scaffold, post = post-implantation, 3M = 3 months follow-up, 6M = 6 months follow-up. *P-value for the comparison between FF-DM and FF-NDM swine, †P-value for the difference between post-procedure and 3M, ‡P-value for the difference between 3M and 6M.
(DOCX)

S2 Table NIRS analysis results. NIRS = Near-infrared spectroscopy, LCBI = lipid core burden index. §P-value for the difference between pre-procedure and 3M. Remaining footnotes and abbreviations are as listed in Table 1.
(DOCX)

S3 Table OCT strut appearance. Percentages are calculated as mean from the total (100%). Footnotes and abbreviations are as listed in Table 1.
(DOCX)

S1 Video 6M OCT pullback of a BVS implanted in a FF-DM swine. OCT imaging at 6M in a FF-DM swine demonstrates a highly heterogeneous appearance of the coverage. The red line in the longitudinal view of the OCT pullback (middle panel) corresponds to the location of the OCT catheter in the 2D pullback (left panel) and 3D pullback (right panel). The asterisk (*) indicates the guidewire artefact, the green line delineates the contour of the lumen area, the stars indicate scaffold struts, the arrowheads lipid, the white circles calcium and the red circle indicates the marker of the scaffold. Word did not find any entries for your table of contents.
(MP4)

S2 Video 6M OCT pullback of BVS in an FF-NDM swine. OCT imaging at 6M in an FF-NDM swine demonstrates a highly heterogeneous appearance of the coverage. The red line in the longitudinal view of the OCT pullback (middle panel) corresponds to the location of the OCT catheter in the 2D pullback (left panel) and 3D pullback (right panel). The green line delineates the contour of the lumen area, the stars indicate scaffold struts, the arrowheads lipid and the white circles indicate calcium.
(MP4)

S1 File Supporting material and methods. Supporting information accompanying the manuscript titled: “Neoatherosclerosis development following bioresorbable vascular scaffold implantation in diabetic and non-diabetic swine coronary arteries”.
(DOC)
