# Supplementary figures and images for: Neoatherosclerosis development following bioresorbable vascular scaffold implantation in diabetic and non-diabetic swine
Source: PLoS One. 2017 Sep 12;12(9):e0183419. doi: 10.1371/journal.pone.0183419 (PMC5595285; doi:10.1371/journal.pone.0183419)

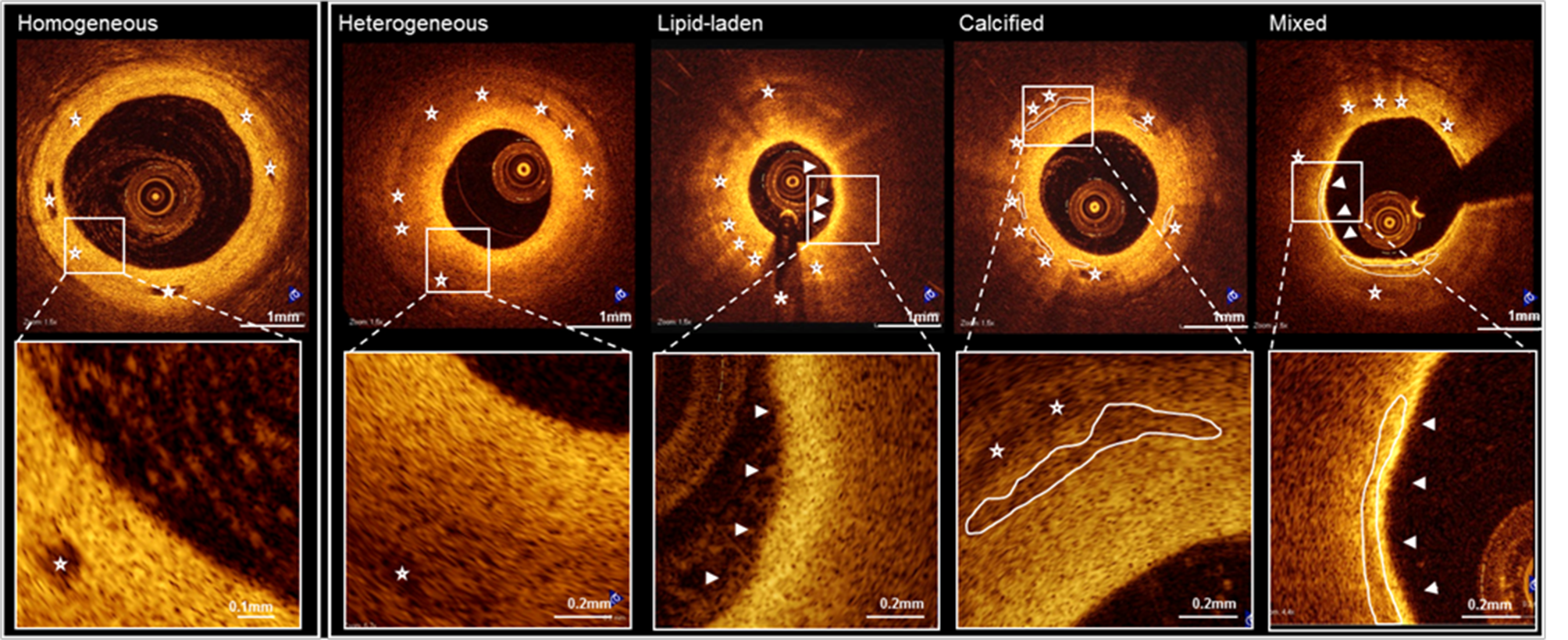

Supplement: S1 Fig — In the top OCT cross-sections of a homogeneous, heterogeneous, lipid-laden, calcified and mixed appearance of the coverage are depicted and on the bottom the magnifications. The ‘open’ stars indicate the black boxes of the scaffold struts at follow-up. The asterix (*) indicates the guide wire artifact. The arrowheads indicate the region containing lipid and the drawn white lines indicate calcified regions. (TIF) [file pone.0183419.s001.tif]

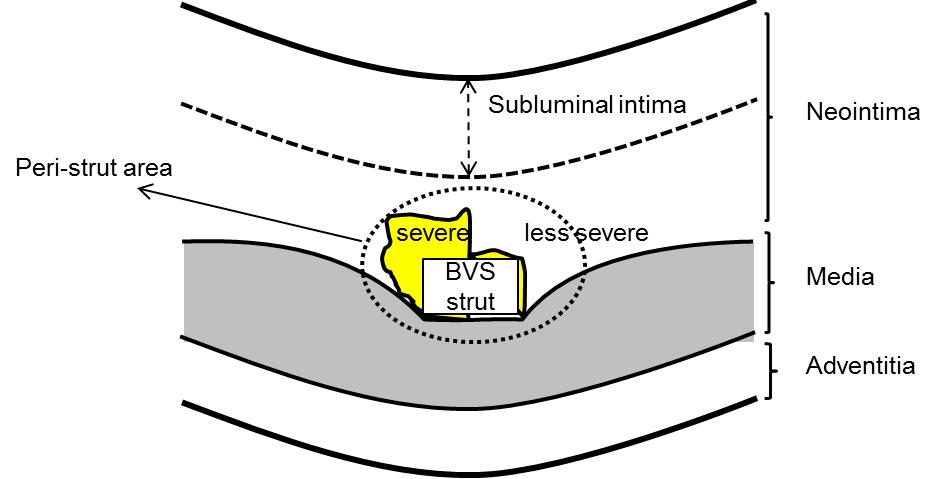

Supplement: S2 Fig — Within the neointima, 2 specific regions were discerned: para-strut neointima, defined as in contact with the struts, and subluminal: located near the lumen. (TIF) [file pone.0183419.s002.tif]

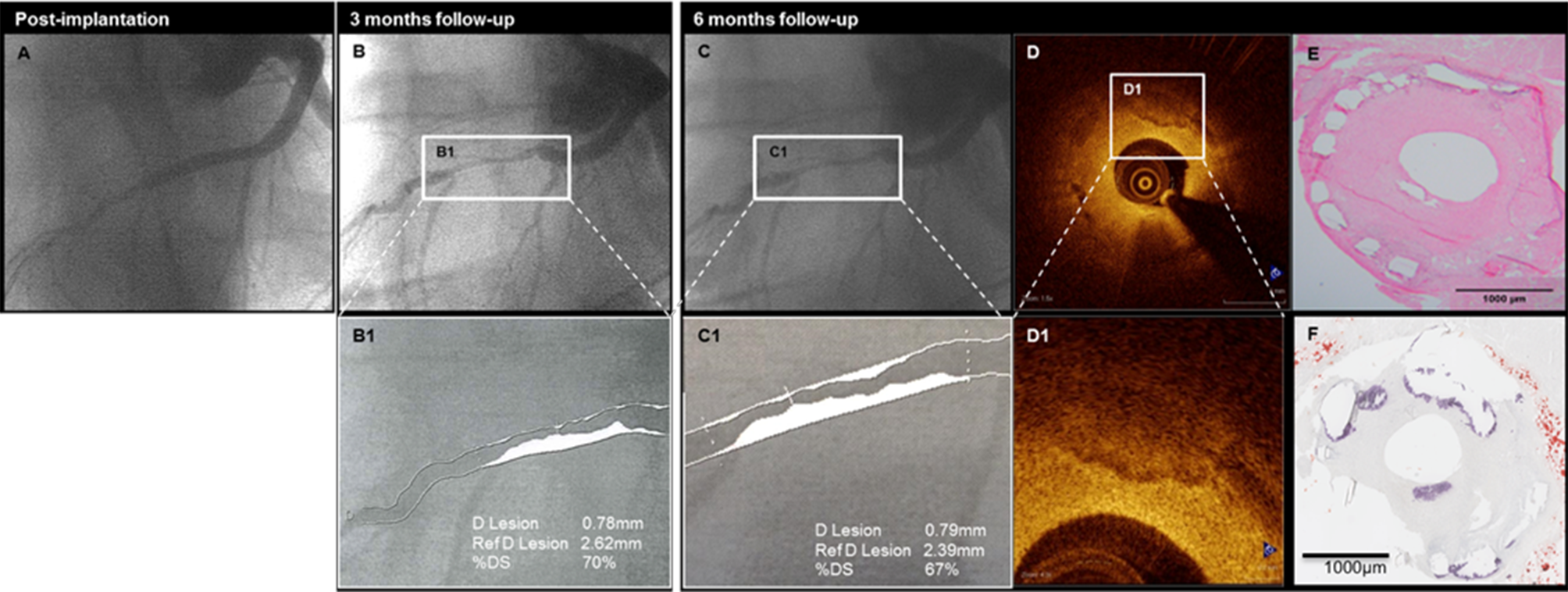

Supplement: S3 Fig — Restenosis of a BVS implanted in an FF-NDM swine at 3M. Coronary angiography post-implantation (A) and at 3M (B) demonstrates a significant lumen loss, with a percentage diameter stenosis (%DS) of 70% which persisted at 6M, (C). At 3M OCT was not performed as the lesion was considered too tight to allow passage of an OCT catheter without risk of causing ischemia and all the potential sequelae thereof. Therefore OCT was restricted to 6M follow-up, the scheduled sacrifice time point. OCT demonstrated a highly heterogeneous neointima (D), which is confirmed by histology demonstrating a large neointimal burden with calcification subluminal and surrounding the struts (E H&E + F, ORO). D Lesion = Diameter of the lesion, Ref D Lesion = Reference diameter. (TIF) [file pone.0183419.s003.tif]

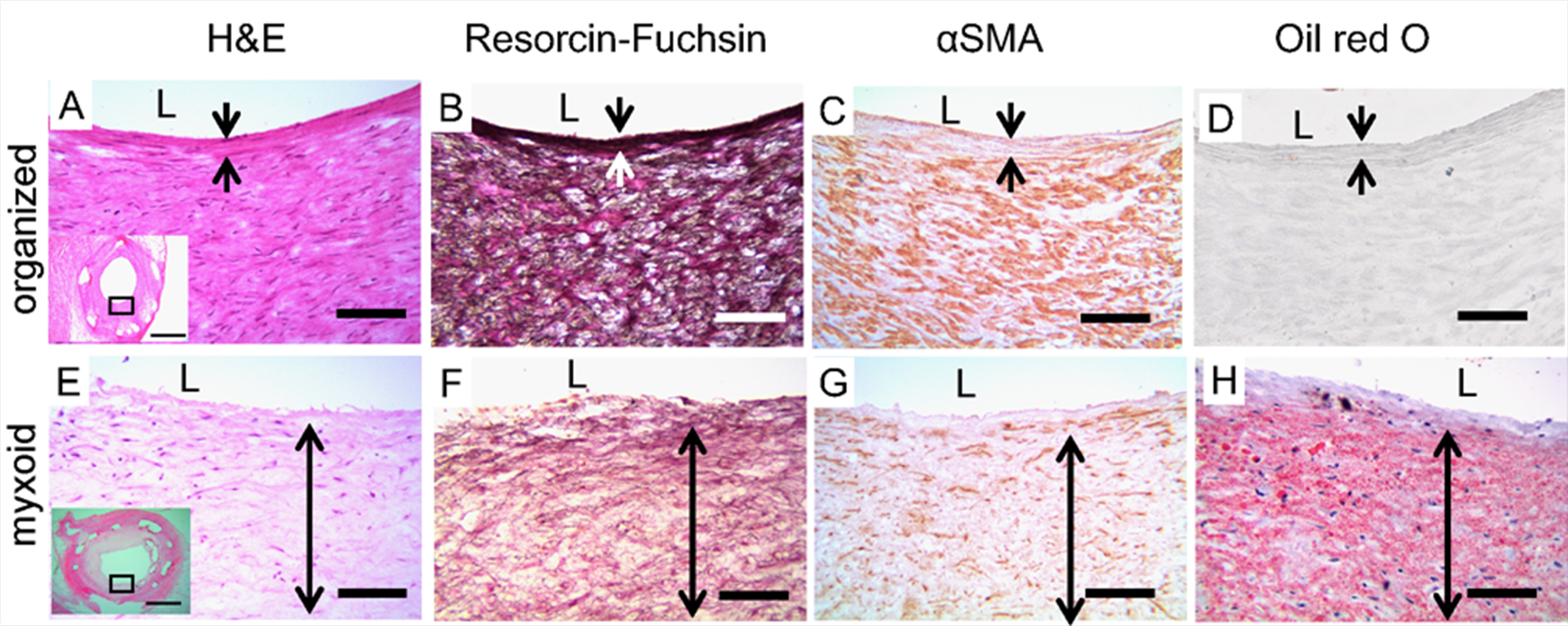

Supplement: S4 Fig — The vessels with well-organized neointimal layers (two arrows in A-D) showed dense elastic fibers (B) with 3 or more layers of αSMA positive cells (C) without lipid accumulation (D). The unorganized neointima showed myxoid degeneration (double arrow in E-H) with disarray and low density of αSMA positive cells (G). In the same area, lipid accumulation was clearly seen (H). αSMA: alpha smooth muscle cell actin, L: lumen, A-D: 3 months DM, E-H: 6 months non-DM, A and E: H&E, B and F: Resorcin-Fuchsin, C and G: αSMA, E and H: Oil red O, Scale bar in A -H: 100 μm, insert bar of A and E: 1000 μm. (TIF) [file pone.0183419.s004.tif]
