# Supplementary material for: Neoatherosclerosis development following bioresorbable vascular scaffold implantation in diabetic and non-diabetic swine
Source: PLoS One. 2017 Sep 12;12(9):e0183419. doi: 10.1371/journal.pone.0183419 (PMC5595285; doi:10.1371/journal.pone.0183419)
Supplement: S1 Table — Normally distributed data are presented as mean ± SD, non-normally distributed data as median (interquartile range). FF-DM = fast-food fed diabetic swine, FF-NDM = fast-food fed non-diabetic swine, QCA = Quantitative coronary angiography, OCT = optical coherence tomography, BVS = bioresorbable vascular scaffold, post = post-implantation, 3M = 3 months follow-up, 6M = 6 months follow-up. *P-value for the comparison between FF-DM and FF-NDM swine, †P-value for the difference between post-procedure and 3M, ‡P-value for the difference between 3M and 6M. (DOCX) [file pone.0183419.s005.docx]

**Supplemental Table 1. Quantitative QCA and OCT analysis results**

|  | **Pre-procedure** | | | **Post-procedure** | | | **3M** | | | **6M** | | | **P†** | | **P‡** | |
| --- | --- | --- | --- | --- | --- | --- | --- | --- | --- | --- | --- | --- | --- | --- | --- | --- |
|  | **FF-DM** | **FF-NDM** | **P*** | **FF-DM** | **FF-NDM** | **P*** | **FF-DM** | **FF-NDM** | **P*** | **FF-DM** | **FF-NDM** | **P*** | **FF-DM** | **FF-NDM** | **FF-DM** | **FF-NDM** |
| **QCA results** |  |  |  |  |  |  |  |  |  |  |  |  |  |  |  |  |
| BVS evaluated | 16 | 14 |  | 16 | 14 |  | 16 | 14 |  | 8 | 12 |  |  |  |  |  |
| Mean scaffold to artery ratio | - | - | - | 1.1±0.1 | 1.1±0.1 | 0.20 | - | - | - | - | - | - | - | - | - | - |
| Mean lumen diameter, mm | 2.79±0.19 | 2.87±0.27 | 0.33 | 3.01±0.11 | 3.03±0.22 | 0.75 | 1.96±0.21 | 1.99±0.39 | 0.82 | 1.87±0.16 | 1.90±0.34 | 0.11 | <0.01 | <0.01 | 0.34 | 0.54 |
| Minimal lumen diameter, mm | - | - | - | - | - | - | 1.61±0.36 | 1.62±0.44 | 0.75 | 1.56±0.26 | 1.69±0.41 | 0.46 | - | - | 0.95 | 0.83 |
| Acute gain, mm | - | - | - | 0.21±0.13 | 0.16±0.13 | 0.22 | - | - | - | - | - | - | - | - | - | - |
| Late loss, mm | - | - | - | - | - | - | 1.05±0.22 | 1.04±0.30 | 0.93 | 1.14±0.20 | 1.10±0.28 | 0.78 | - | - | 0.24 | 0.65 |
| **OCT results** |  |  |  |  |  |  |  |  |  |  |  |  |  |  |  |  |
| BVS evaluated, n | 16 | 14 |  | 16 | 14 |  | 16 | 14 |  | 7 | 12 |  | 16 | 14 | 7 | 12 |
| Mean discernible struts per cross-section, n | - | - | - | 8.16±1.57 | 8.25±0.69 | 0.90 | 7.94±1.07 | 8.05±0.91 | 0.72 | 6.58±1.18 | 6.65±1.78 | 0.95 | 0.66 | 0.53 | 0.01 | <0.01 |
| Mean lumen area, mm^2^ | 7.23±1.42 | 6.79±1.37 | 0.46 | 8.26±0.57 | 8.27±0.52 | 0.99 | 3.07±0.59 | 3.25±1.12 | 0.64 | 3.12±0.46 | 2.85±1.06 | 0.55 | <0.01 | <0.01 | 0.92 | <0.01 |
| Mean lumen diameter, mm | 3.01±0.30 | 2.92±0.31 | 0.46 | 3.23±0.12 | 3.24±0.10 | 0.94 | 1.95±0.20 | 1.99±0.38 | 0.79 | 1.97±0.15 | 1.86±0.37 | 0.45 | <0.01 | <0.01 | 0.97 | <0.01 |
| Minimal lumen diameter, mm | 2.57±0.42 | 2.53±0.26 | 0.74 | 2.76±0.31 | 2.83±0.28 | 0.55 | 1.53±0.32 | 1.62±0.27 | 0.29 | 1.58±0.19 | 1.48±0.29 | 0.34 | <0.01 | <0.01 | 0.71 | 0.01 |
| Mean scaffold area, mm^2^ | - | - | - | 8.29±0.55 | 8.31±0.52 | 0.92 | 7.45±0.36 | 7.32±0.84 | 0.71 | 7.17±0.36 | 7.16±0.94 | 0.88 | <0.01 | <0.01 | 0.19 | 0.35 |
| Mean scaffold diameter, mm | - | - | - | 3.24±0.11 | 3.25±0.10 | 0.85 | 3.07±0.08 | 3.04±0.18 | 0.70 | 3.02±0.08 | 3.01±0.20 | 0.92 | <0.01 | <0.01 | 0.19 | 0.33 |
| Mean prolapse area, mm^2^ [n] | - | - | - | 0.22  (0.04;0.55) [6] | 0.29 (0.20;0.35) [10] | 0.51 | - | - | - | - | - | - | - | - | - | - |
| Distal reference lumen area, mm^2^ | 5.60±1.66 | 5.20±1.51 | 0.59 | 4.38±1.27 | 4.59±1.05 | 0.66 | 4.50±1.32 | 4.59±1.19 | 0.81 | 4.50±0.88 | 4.01±1.99 | 0.29 | 0.34 | 0.82 | 0.91 | 0.02 |
| Proximal reference lumen area, mm^2^ | 8.97±1.99 | 8.71±1.88 | 0.72 | 8.34±194 | 8.26±2.02 | 0.91 | 8.56±2.56 | 8.03±2.03 | 0.54 | 8.42±1.29 | 6.45±2.80 | 0.15 | 0.90 | 0.60 | 0.62 | 0.04 |

Normally distributed data are presented as mean ± SD, non-normally distributed data as median (interquartile range). FF-DM = fast-food fed diabetic swine, FF-NDM = fast-food fed non-diabetic swine, QCA = Quantitative coronary angiography, OCT = optical coherence tomography, BVS = bioresorbable vascular scaffold, post = post-implantation, 3M = 3 months follow-up, 6M = 6 months follow-up. *P-value for the comparison between FF-DM and FF-NDM swine, †P-value for the difference between post-procedure and 3M, ‡P-value for the difference between 3M and 6M
