# Supplementary material for: Neoatherosclerosis development following bioresorbable vascular scaffold implantation in diabetic and non-diabetic swine
Source: PLoS One. 2017 Sep 12;12(9):e0183419. doi: 10.1371/journal.pone.0183419 (PMC5595285; doi:10.1371/journal.pone.0183419)
Supplement: S2 Table — NIRS = Near-infrared spectroscopy, LCBI = lipid core burden index. §P-value for the difference between pre-procedure and 3M. Remaining footnotes and abbreviations are as listed in Table 1. (DOCX) [file pone.0183419.s006.docx]

**Supplemental Table 2. NIRS analysis results**

|  | **Pre-procedure** | | | **3M** | | | **6M** | | | **P§** | | **P‡** | |
| --- | --- | --- | --- | --- | --- | --- | --- | --- | --- | --- | --- | --- | --- |
|  | **FF-DM** | **FF-NDM** | **P*** | **FF-DM** | **FF-NDM** | **P*** | **FF-DM** | **FF-NDM** | **P*** | **FF-DM** | **FF-NDM** | **FF-DM** | **FF-NDM** |
| BVS evaluated, n | 15 | 11 |  | 15 | 11 |  | 6 | 8 |  |  |  |  |  |
| LCBI | 0.00  (0.00;5.00) | 0.00  (0.00;1.00) | 0.34 | 2.00  (0.00;15.50) | 0.00  (0.00;3.00) | 0.69 | 17.50  (9.75;26.00) | 6.50 (0.00;47.25) | 0.49 | 0.88 | 0.50 | 0.80 | 0.18 |

NIRS = Near-infrared spectroscopy, LCBI = lipid core burden index. §P-value for the difference between pre-procedure and 3M. Remaining footnotes and abbreviations are as listed in Table 1
