# Supplementary material for: Neoatherosclerosis development following bioresorbable vascular scaffold implantation in diabetic and non-diabetic swine
Source: PLoS One. 2017 Sep 12;12(9):e0183419. doi: 10.1371/journal.pone.0183419 (PMC5595285; doi:10.1371/journal.pone.0183419)
Supplement: S3 Table — Percentages are calculated as mean from the total (100%). Footnotes and abbreviations are as listed in Table 1. (DOCX) [file pone.0183419.s007.docx]

**Supplemental Table 2. OCT strut appearance**

|  |  | **3M** | |  | | **6M** | |  | | **P‡** | |
| --- | --- | --- | --- | --- | --- | --- | --- | --- | --- | --- | --- |
|  |  | **FF-DM** | **FF-NDM** | | **P*** | **FF-DM** | **FF-NDM** | | **P*** | **FF-DM** | **FF-NDM** |
| BVS evaluated, n | | 16 | 12 | |  | 7 | 12 | |  |  |  |
| Mean discernible struts per cross-section, n | | 8±1 | 8±1 | | 0.72 | 7±1 | 6±2 | | 0.79 | 0.01 | <0.01 |
| **Box classification** | |  |  | |  |  |  | |  |  |  |
| Preserved box, % | | 79±8 | 81±11 | | 0.63 | 77±13 | 68±17 | | 0.30 | 0.68 | 0.05 |
| Open box, % | | 1.0±1.5 | 1.1±2.3 | | 0.88 | 0.3±0.9 | 1.4±1.3 | | <0.01 | 0.29 | 0.50 |
| Dissolved black box, % | | 20±8 | 18±12 | | 0.61 | 22±14 | 31±17 | | 0.35 | 0.53 | 0.06 |
| Dissolved bright box, % | | 0 | 0 | | - | 0 | 0 | | - | - |  |

Percentages are calculated as mean from the total (100%). Footnotes and abbreviations are as listed in Table 1
