# Supplementary material for: Neoatherosclerosis development following bioresorbable vascular scaffold implantation in diabetic and non-diabetic swine
Source: PLoS One. 2017 Sep 12;12(9):e0183419. doi: 10.1371/journal.pone.0183419 (PMC5595285; doi:10.1371/journal.pone.0183419)
Supplement: S1 File — Supporting information accompanying the manuscript titled: “Neoatherosclerosis development following bioresorbable vascular scaffold implantation in diabetic and non-diabetic swine coronary arteries”. (DOC) [file pone.0183419.s010.doc]

**Neoatherosclerosis development following bioresorbable vascular scaffold implantation in diabetic and non-diabetic swine coronary arteries**

**Short Title:** Neoatherosclerosis in BVS in DM and NDM swine

Nienke S. van Ditzhuijzen, Msc^1^, Mie Kurata, MD, PhD^1^, Mieke van den Heuvel, MD^1^, Oana Sorop, PhD^1^, Richard W.B. van Duin^1^, Ilona Krabbendam-Peters^1^, Jurgen Ligthart, RT^1^, Karen Witberg, CCRN^1^, Magdalena Murawska, PhD^2^, Brett Bouma, PhD^4^, Martin Villiger, PhD^4^, Hector M. Garcia-Garcia, MD, PhD^3^, Patrick W. Serruys, MD, PhD^3^, Felix Zijlstra, MD, PhD^1^, Gijs van Soest, PhD^1^, Dirk-Jan Duncker, MD, PhD^1^, Evelyn Regar, MD, PhD^1, 5^, Heleen M.M. van Beusekom, PhD^1^

*^1^Department of Cardiology, Thoraxcenter, Cardiovascular Research school COEUR, ^2^Department of Biostatistics, Erasmus University Medical Center, Rotterdam, The Netherlands, ^3^Cardialysis B.V., Rotterdam, The Netherlands, ^4^Wellman Center for Photomedicine, Massachusetts General Hospital, Harvard Medical School, Boston, MA, USA**, ^5^Dept. of Cardiovascular Surgery, University Hospital Zurich, Switzerland*

**- Supporting material and methods -**

**Total word count**: 2353; **Supporting Tables**: 3, **Supporting Figures**: 4; **Supporting Videos**: 2

Corresponding author: h.vanbeusekom@erasmusmc.nl

**Supporting Material and Methods**

**Scaffold implantation procedure**

The first generation Absorb Bioresorbable Vascular Scaffold (BVS), revision 1.0, was studied in the Absorb Cohort A clinical trial, as well as in an experimental study, evaluating degradation of the scaffold up to 4 years in swine. The BVS revision 1.0 was designed with a crossing profile of 1.4mmm with circumferential hoops and a strut thickness of 150micrometer, directly joined or linked by straight bridges, containing a Poly-D,L-lactide acid (PDLLA) coating, eluting everolimus. The second generation Absorb BVS, revision 1.1, has a similar polymeric coating, strut thickness and drug-coating, however, it consists of in-phase zigzag hoops linked by bridges that allow for more uniform strut distribution, reducing maximum circular unsupported surface area and providing more uniform vessel wall support and drug transfer.

In the current study, nine months after its start, 32 single 3.0 x 18.0mm Bioresorbable Vascular Scaffold (BVS) revision 1.1 (Abbott Vascular, Santa Clara, CA) were implanted via carotid access under the guidance of quantitative coronary angiography (QCA) and optical coherence tomography (OCT). In case of major discrepancy (>0,5mm in diameter lumen) between angiography and OCT, OCT was considered the gold standard for selection of BVS position and sizing. To achieve a stent to artery ratio of ≥ 1.1:1 delivery balloons were inflated with a mean pressure of 12±2 atm according to the measured lumen diameter and the compliance chart of the balloons.

**Intracoronary imaging and sacrifice at 3M and 6M**

After an overnight fast, the swine were sedated using ketamine/ midazolam (20 mg/kg / 1 mg/kg i.m.) and atropine (1mg/30kg i.m.). Induction of anesthesia was performed using thiopental (15 mg/kg i.v.). Vascular access was obtained with an 8F vascular sheath in the carotid artery, 10.000 IU heparin was administered initially and thereafter 5000 IU of heparin was administered every hour. The swine were connected to a ventilator that administered a mixture of oxygen and nitrous oxide (1:2 [vol/vol]) and anesthesia was maintained using 0.5-2.5 vol% isoflurane. Antibiotic prophylaxis was administered by an intramuscular injection of 8 mL of 200 mg/mL procaine-benzylpenicillin and 250 mg/mL streptomycin. Sacrifice of the swine was performed by an intravenous injection of pentobarbital euthanasia solution (100mg/kg).

Due to the increased weight of the swine at 6M, pentobarbital (5mg/ kg i.m.) instead of ketamine/ midazolam was used for sedation after an overnight fast. Induction of anesthesia was performed as described for the 3 months follow-up but anesthesia was maintained using pentobarbital (5mg/ kg i.m.) and sacrifice of the swine was performed by an intravenous injection of pentobarbital euthanasia solution (100mg/kg).

**QCA analysis**

S/A ratio was calculated as the maximum balloon diameter during implantation of the scaffold divided by the mean lumen diameter pre-implantation. Acute gain was defined by the difference between mean lumen diameter before scaffold implantation and immediately after scaffold implantation. Late lumen loss was defined by the difference between mean lumen diameter immediately post-implantation and at 3M or 6M.

**OCT analysis**

OCT analysis of the serial data was performed off-line according to previously published methodology. Before OCT analysis, the Z-offset was checked in all pullbacks and corrected if necessary. The region of interest was defined as the scaffolded segment plus 5mm proximal and 5mm distal from the scaffolded segment. Pre-implantation we measured mean lumen area (LA), EEM area and mean plaque burden (%PB). Contours of the lumen area were obtained with a semi-automated detection algorithm using the LightLab proprietary software for off-line OCT analysis (St. Jude medical, Westford, MA, USA) and additional manual corrections were performed if necessary. Contours of the EEM area were obtained to calculate the mean % plaque burden and were measured by the *multiple point trace* function of the offline OCT analysis software. Mean plaque burden (%PB) was calculated as [(EEM area – LA)/ EEM area] * 100.

Post-implantation and at 3M and 6M we measured mean lumen area, scaffold area (SA) and incomplete strut apposition (ISA) area. The lumen area can be imaged because of the translucency of the polymeric struts, and is delineated by the endoluminal contour of the vessel wall. Scaffold area is measured by joining the middle point of the abluminal side of the black core of the struts. Incomplete strut apposition (ISA) is defined as a clear separation, by a contrast-filled gap, between the back (abluminal) side of the strut and the vessel wall and ISA area is delineated by the area between the abluminal side of the frame border of the malapposed strut and the endoluminal contour of the vessel wall. In case of complete strut apposition immediately post-implantation, scaffold area is identical to lumen area. In case of prolapse protruding between struts into the lumen immediately post-implantation, the prolapse area was calculated as difference between the lumen area and the scaffold area. A thrombus was identified as an intra-luminal mass, with no direct continuity with the surface of the vessel wall or as a highly backscattered luminal protrusion in continuity with the vessel wall and resulting in signal-free shadowing.

Coverage area (CA) and coverage thickness (CT) were calculated at 3M and 6M. Lumen area is drawn by following the endoluminal contour of the neointima between and on top of the struts. Scaffold area is measured as post-implantation. Due to high heterogeneity of the coverage at follow-up, the contours of the black core area of the scaffold struts could not always be clearly identified. In case the contours of the black core areas could not be traced, the scaffold area contour could be interpolated from a cross-section distal or proximal to the cross-section selected for analysis.

From previous animal studies we know that each polymeric strut is ultimately replaced by proteoglycan and connective tissue, therefore, in order to have a representative measure of the coverage of the lesion treated with BVS, coverage area was defined as [scaffold area – lumen area]. To evaluate whether the clearly visible struts were covered or uncovered, the coverage thickness (CT) of these struts was measured as the length between the abluminal border of the black core area of the strut and the lumen contour following the center of gravity of the lumen area. The threshold for coverage is 30 microns of the endoluminal light backscattering frame of the strut.

The morphology the scaffold coverage – defined as [SA – LA], as has been described previously – was described per OCT cross-section as homogeneous or heterogeneous. Homogeneous coverage is defined as coverage that contains uniform optical properties and does not demonstrate focal variations in backscattering pattern and heterogeneous coverage as coverage that contains focally changing optical properties and shows various backscattering patterns. The heterogeneous coverage was furthermore classified into lipid-laden, calcified or mixed. Lipid-laden coverage was defined as coverage clearly containing signal poor regions with diffusely delineated borders, calcified as coverage clearly containing signal poor regions with sharply delineated borders either present around the struts or subluminal, and mixed coverage was defined as coverage containing both a lipid-laden and calcified appearance (**S2 Fig**). The presence of calcium was furthermore classified as a) surrounding the struts, defined as calcium located in the abluminal half of the neointima (towards the scaffold struts), and b) subluminal, defined as calcium located in the adluminal half of the neointima (in contact with the luminal arterial wall).

**NIRS analysis**

The NIRS catheter was advanced distally in the coronary artery over a 0.14inch guide wire through an 8F guiding catheter and pulled back automatically with a pullback speed of 0.5mm/sec. Quantitative NIRS analysis was performed in the region of interest, thus scaffolded segment + 5mm proximal and distal, that was matched between all time-points, using the off-line LipiScan analyzer software. A quantitative lipid-core burden index (LCBI) is provided as a summary metric of the LCP over the scaffolded segment and is computed as a fraction of valid pixels within the scanned region that exceed an LCP probability of 0.6, multiplied by 1000. Moreover, the LCP(+) signal is indicated by yellow in the chemogram when the algorithm indicates there is a high probability of the presence of an LCP of interest.

**Ex-vivo degradation analysis**

For the GPC-analysis, the initial mass was determined for all scaffolds. As a control, weight-average molecular weight (Mw), number-average molecular weight (Mn) and polydispersity index [PDI = Mw/ Mn] were determined in 5 scaffolds that were not implanted but were from the same batch as the implanted scaffolds. After sacrifice of the swine at 3M and 6M, Mw, Mn, PDI and polymer mass loss [% mass loss = (Initial mass [T=0] – Found mass) / Initial mass [T=0] * 100] were determined for GPC-analysis.

**Ex-vivo histological analysis**

BVS designated for histology were embedded in tissue-tek (Sakura Finetek, Japan) and frozen in N_2_-cooled isopentane (n=17). All tissue sections were stained by Hematoxylin-Eosin (HE) as an overview stain, Resorcin-Fuchsin for elastin, Alcian-Blue for low pH and proteoglycans, Oil-red-O (ORO) for fat, Picrosirius Red (PSR) for collagen, von Kossa for calcium, and immunohistochemistry for smooth muscle cells SMA, clone 1A4, Dako, the Netherlands) and leukocytes (CD45, clone MCA 1447, AbD Serotec, UK).

*Neointimal healing and organization* Struts covered with neointima without remnant thrombus or fibrin, were defined as healed. Luminal organization was scored as 2 if present in more than 50% of the circumference with 3 or more layers of SMA positive cells, as 1 if present in less than 50% of the circumference or as 0 if not present at all. Signs of myxoid degeneration were noted.

*Neointimal collagen distribution* If PSR staining was diminished in bright field and absent under polarized light, tissue was stratified as collagen poor.

*Injury and inflammation* Injury was scored on Elastin stained sections (Schwartz score). Inflammation around struts was scored using HE, ranking from 0 (no inflammatory cells) to 3 (>20 inflammatory cell per strut) (Cheneau score).

*Neointimal calcification* Subluminal calcification was scored as present or not. Para-strut calcification was calculated as % calcification-positive struts regardless of severity (100x(calcified struts/total struts)). Severity of para-strut calcification was scored as rank 0 for none, rank 1 for partial rims of circumstrut calcium, rank 2 if extending beyond a rim, and rank 3 when extensive and connecting struts and given as the maximum score per scaffold.

Injury, inflammation and calcification scores were calculated per section as the sum of strut scores divided by the number of struts. Mean scores per segment were averaged per scaffold. Subluminal calcifications and collagen poor areas are given as presence per scaffold.

*Lipid accumulation* Was documented as the proportion of ORO-positivity within the neointimal area.

**References**

1. Onuma Y, Serruys PW, Perkins LE, Okamura T, Gonzalo N, Garcia-Garcia HM, et al. Intracoronary optical coherence tomography and histology at 1 month and 2, 3, and 4 years after implantation of everolimus-eluting bioresorbable vascular scaffolds in a porcine coronary artery model: an attempt to decipher the human optical coherence tomography images in the ABSORB trial. Circulation. 2010;122(22):2288-300. Epub 2010/10/27. doi: CIRCULATIONAHA.109.921528 [pii]

10.1161/CIRCULATIONAHA.109.921528. PubMed PMID: 20975003.

2. Okamura T, Garg S, Gutierrez-Chico JL, Shin ES, Onuma Y, Garcia-Garcia HM, et al. In vivo evaluation of stent strut distribution patterns in the bioabsorbable everolimus-eluting device: an OCT ad hoc analysis of the revision 1.0 and revision 1.1 stent design in the ABSORB clinical trial. EuroIntervention. 2010;5(8):932-8. Epub 2010/06/15. doi: EIJV5I8A157 [pii]

10.4244/. PubMed PMID: 20542778.

3. Serruys PW, Onuma Y, Ormiston JA, de Bruyne B, Regar E, Dudek D, et al. Evaluation of the second generation of a bioresorbable everolimus drug-eluting vascular scaffold for treatment of de novo coronary artery stenosis: six-month clinical and imaging outcomes. Circulation. 2010;122(22):2301-12. Epub 2010/11/26. doi: CIRCULATIONAHA.110.970772 [pii]

10.1161/CIRCULATIONAHA.110.970772. PubMed PMID: 21098436.

4. van Ditzhuijzen NS, Karanasos, A., Bruining, N., van den Heuvel, M., Sorop, O., Ligthart, J., Witberg, K., Garcia-Garcia, H.M., Zijlstra, F., Duncker, D.J., van Beusekom, H.M.M., Regar, E. The impact of Fourier-Domain optical coherence tomography catheter induced motion artefacts on quantitative measurements of a PLLA-based bioresorbable scaffold. Accepted in Int J Cardiovasc Imaging. 2014. doi: 10.1007/s10554-014-0447-3.

5. Prati F, Guagliumi G, Mintz GS, Costa M, Regar E, Akasaka T, et al. Expert review document part 2: methodology, terminology and clinical applications of optical coherence tomography for the assessment of interventional procedures. Eur Heart J. 33(20):2513-20. PubMed PMID: 22653335.

6. Kume T, Akasaka T, Kawamoto T, Ogasawara Y, Watanabe N, Toyota E, et al. Assessment of coronary arterial thrombus by optical coherence tomography. The American journal of cardiology. 2006;97(12):1713-7. PubMed PMID: 16765119.

7. Brugaletta S, Radu MD, Garcia-Garcia HM, Heo JH, Farooq V, Girasis C, et al. Circumferential evaluation of the neointima by optical coherence tomography after ABSORB bioresorbable vascular scaffold implantation: can the scaffold cap the plaque? Atherosclerosis. 2012;221(1):106-12. doi: S0021-9150(11)01153-1 [pii]

10.1016/j.atherosclerosis.2011.12.008. PubMed PMID: 22209268.

8. Kang SJ, Mintz GS, Akasaka T, Park DW, Lee JY, Kim WJ, et al. Optical coherence tomographic analysis of in-stent neoatherosclerosis after drug-eluting stent implantation. Circulation. 2011;123(25):2954-63. Epub 2011/06/08. doi: 10.1161/CIRCULATIONAHA.110.988436. PubMed PMID: 21646494.

9. van Ditzhuijzen NS, van Beusekom HM, Ligthart JM, Regar E. Invasive imaging of the coronary atherosclerotic plaque. Minerva Cardioangiol. 2012;60(3):305-29. Epub 2012/06/02. doi: R05123278 [pii]. PubMed PMID: 22653045.

10. Schwartz RS, Huber KC, Murphy JG, Edwards WD, Camrud AR, Vlietstra RE, et al. Restenosis and the proportional neointimal response to coronary artery injury: results in a porcine model. J Am Coll Cardiol. 1992;19(2):267-74. PubMed PMID: 1732351.
